# The care provided to black-skinned children and adolescents with mental health problems in the intersection between gender and race*

**DOI:** 10.1590/1518-8345.6058.3942

**Published:** 2023-06-19

**Authors:** Luciane Régio, Sônia Barros, Caroline Ballan, Carla Aguiar, Bruna de Paula Candido, Márcia Aparecida Ferreira de Oliveira

**Affiliations:** 1 Universidade de São Paulo, Escola de Enfermagem, São Paulo, SP, Brasil.; 2 Faculdade de Ciências Médicas da Santa Casa, Curso de Enfermagem, São Paulo, SP, Brasil.; 3 Universidade de São Paulo, Instituto de Estudos Avançados, São Paulo, SP, Brasil.

**Keywords:** Mental Health, Gender, Race, Children, Adolescents, Community Mental Health Centers, Salud Mental, Género, Raciales, Niños, Adolescentes, Centros Comunitarios de Salud Mental, Saúde Mental, Gênero, Raça, Crianças, Adolescentes, Centros Comunitários de Saúde Mental

## Abstract

**Objective:**

to characterize the sociofamily profile of black-skinned children and adolescents with mental health problems and to intersectionally describe who assumes responsibility for their care.

**Method:**

a descriptive and exploratory study with a quantitative approach, developed in the Psychosocial Care Center for Children and Adolescents from the North region of the municipality of São Paulo. The data were collected from 47 family members of black-skinned children and adolescents, using a script with predefined variables submitted to statistical analysis.

**Results:**

a total of 49 interviews were conducted: 95.5% women with a mean age of 39 years old, 88.6% mothers and 85.7% black-skinned. Family income comes from wages for all the male caregivers and for 59% of the women. Among the black-skinned female caregivers, 25% live in their own house, whereas this percentage is 46.2% among the brown-skinned ones. Of all the caregivers, 10% have a job, 20% live in transferred properties, 35% in houses of their own and 35% in rented places. The social support network is larger among white-skinned people (16.7%), followed by brown-skinned (3.8%), and absent among black-skinned individuals (0%).

**Conclusion:**

those responsible for the care of black-skinned children and adolescents monitored by the CAPS-IJ are almost entirely women, black-skinned (black or brown) “mothers or grandmothers”, with unequal access to education, work and housing, constitutional social rights in Brazil.

## Abstract (es)

**Objetivo:**

caracterizar el perfil sociofamiliar de niños y adolescentes negros con problemas de salud mental y describir desde un enfoque interseccional quién es responsable de su cuidado.

**Método:**

estudio exploratorio descriptivo de enfoque cualitativo, llevado a cabo en un Centro de Atención Psicosocial Infantil y Juvenil de la región norte del municipio de São Paulo. La recolección de datos se realizó con 47 familiares de niños y adolescentes negros, con el soporte de un guion con variables predefinidas, sometidas a análisis estadístico.

**Resultados:**

se realizaron 49 entrevistas, 95,5% con mujeres, con edad promedio de 39 años, 88,6% madres, 85,7% negras. La renta familiar procede del salario para el 100% de los cuidadores hombres y el 59% de las mujeres. Entre las cuidadoras negras, 25% tienen casa propia y, entre las pardas, 46,2%. Del total de cuidadores, el 10% vive en casas ocupadas, el 20% vive en viviendas cedidas, el 35% tiene casa propia y el 35% alquila. La red de contención social es mayor entre los blancos (16,7%), seguidos por los pardos (3,8%) y está ausente entre los negros (0%).

**Conclusión:**

las responsables por el cuidado de niños y e adolescentes negros atendidos en el CAPSij, son, casi en su totalidad mujeres, “madres o abuelas” negras (o mulatas), con acceso desigual a educación, trabajo y vivienda, derechos sociales constitucionales en Brasil.

## Abstract (pt)

**Objetivo:**

caracterizar o perfil sociofamiliar de crianças e adolescentes negros com problemas de saúde mental e descrever interseccionalmente quem se responsabiliza por seus cuidados.

**Método:**

estudo descritivo exploratório de abordagem quantitativa, desenvolvido em um Centro de Atenção Psicossocial Infantojuvenil da região norte do município de São Paulo. Os dados foram coletados com 47 familiares de crianças e adolescentes negros, utilizando um roteiro com variáveis pré-definidas, submetidas à análise estatística.

**Resultados:**

foram realizadas 49 entrevistas, sendo 95,5% com mulheres, média de idade de 39 anos, 88,6% mães, 85,7% negras. A renda familiar é proveniente de salário, para 100% dos cuidadores homens e para 59% das mulheres. Dentre as cuidadoras pretas, 25% possuem casa própria, sendo que, dentre as pardas, 46,2%. Do total de cuidadores, 10% vivem em condições de ocupação, 20% habitam moradias cedidas, 35% casas próprias e 35% alugadas. A rede social de suporte é maior entre os brancos (16,7%), seguido pelos pardos (3,8%) e ausente entre os pretos (0%).

**Conclusão:**

as responsáveis pelo cuidado de crianças e adolescentes negros acompanhados pelo CAPSij, são na quase totalidade mulheres, “mães ou avós” negras (pretas ou pardas), com acesso desigual à educação, trabalho e moradia, direitos sociais constitucionais no Brasil.

**Highlights:**

**(1)** The socio-family profile exerts an influence on children’s and adolescents’ health-disease process.

**(2)** Inequalities in terms of gender, race/skin color, ethnicity and social class are mental health determinants.

**(3)** Intersectionality as an analytical category to improve care in freedom within the SUS.

## Introduction

Mental health quality is directly related to access to and guarantee of human rights because the social, economic, political, cultural, geographic and ethnic-racial conditions, intersected, exert impacts on people’s lives^(1)^. According to the World Health Organization’s Global Strategy for the Health of Women, Children and Adolescents (2016-2030), precarious living conditions significantly interfere with the health and well-being of these population segments, with inequalities in physical, cognitive, psychological and/or or socioemotional development^(2)^.

In Brazil, with the 1988 Constitution and the Unified Health System (*Sistema Único de Saúde*, SUS), health policies focused on trying to reduce gender, race, age, social class, ethnicity and geographic inequalities^(3 4)^. The agenda of mental health care for children and adolescents was included in the II National Conference on Mental Health in 1992, and the Psychosocial Care Centers for Children and Adolescents (*Centros de Atenção Psicossocial Infantojuvenil*, CAPS-IJs) were established by Ordinance No. 336/2002^(5)^.

The United Nations (UN) Convention on Children’s Rights, ratified by all world countries (except USA), addresses children’s rights to mental health. Children with impairments/disabilities frequently face marginalization and discrimination, and their vulnerabilities are compounded by poverty, social and humanitarian isolation, emergencies, and lack of institutional, family and social support networks^(6)^.

Historically, health care for the black-skinned population has, and still is, quantitatively and qualitatively inferior than for white-skinned people^(7)^. The National Policy for Comprehensive Health of the Black-Skinned Population (*Política Nacional de Saúde Integral da População Negra*, PNSIPN) is based on combating these inequalities and institutional racism in health services^(8 9)^.

Regarding the profile of people who use Psychosocial Care Centers (CAPS), a literature review pointed to the scarcity of Brazilian research studies using the “race/skin color” variable, a fact that delays the formulation of adequate public policies to face social and racial inequalities. The recommendation is for the variable to be used in research studies as an analysis category, as it is an important social marker to contribute to practices and policies aimed at ensuring rights^(10 11)^.

Regarding the family configuration of children and adolescents monitored by mental health services specialized in this population segment, a number of studies indicate that the majority belong to nuclear families and live with their parents^(12 13)^. One study points out that mothers are the main caregiver of children and adolescents with mental health problems and that, in cases where they were responsible for the family income, they were also assigned the care of the children; on the other hand, when providers of these families, the fathers were not responsible for the care of the children^(12)^.

Family constitution, financial resources, the social support network and the individual resources of the caregivers exert an influence on children’s and adolescents’ health/disease process^(14)^. Care involves activities such as washing, cooking, cleaning, organizing the routine, establishing affective bonds, ensuring protection, health, offering affection and understanding, as well as in the development of physical, social and/or emotional capacities. This is social reproduction work fundamental for the well-being of societies, communities and consequent functioning of the economy, historically carried out by women, unpaid in the home space, or in an important work front when it passes from the family to the market^(15)^.

The physical and emotional burden imposed by the care work causes changes in the families’ routines, habits and customs. These are processes that require organization, with repercussions in the financial, social, occupational and personal dimensions. The starting point is the way in which we respond to human life sustainability needs perpetuates the gender, race, and class inequalities^(16)^. In addition to the fact that, in order to provide care to black-skinned children and adolescents with mental health problems, it is necessary to take care of those who care for them. The objective of this study was to characterize the sociofamily profiles of black-skinned children and adolescents with mental health problems, as well as to intersectionally describe the people responsible for their care.

## Method

### Study design

A descriptive and exploratory study with a quantitative approach with the intention of allowing for the sociodemographic understanding of historical and social processes, and supporting the formulation of care practices and public policies that guarantee equality and integrality for universal access to health.

### Setting

A community-based free care device, strategic in the Psychosocial Care Network (*Rede de Atenção Psicossocial*, RAPS), a Psychosocial Care Center for Children and Adolescents (CAPS-IJ) in the city of São Paulo/SP, Brazil, of indirect administration and in a management contract with a Social Health Organization (SHO). SHOs are non-state public property modalities, constituted by non-profit civil associations, and must be directly oriented towards serving the public interest^(17)^.

The CAPS-IJ is located in the North Area of the city; its coverage area is limited to the Freguesia/Brasilândia Technical Health Supervision Office, made up of the administrative districts Freguesia do Ó, where 30% of the population is black-skinned, and Brasilândia, where 50% of the residents are black-skinned. It is worth pointing out that the device is located in the noble and white area of the Health Supervision, away from the vulnerable communities, home to the majority of the black- and brown-skinned people monitored^(18)^.

The Brasilândia CAPS-IJ is a reference for a population of 420,000 inhabitants; it monitors children and adolescents with severe and persistent mental health problems and using crack and other drugs, open and of a community and territorial nature^(19)^.

### Population

Family members of black- and brown-skinned children and adolescents undergoing monitoring in the Brasilândia CAPS-IJ.

### Selection criteria

The following inclusion criteria were adopted: being a family member of black- or brown-skinned children and adolescents undergoing monitoring for at least one month and with preserved comprehension and communication skills. The exclusion criterion corresponded to medical charts with no monitoring activities in the last month.

### Study variables

The script consisted of the following variables: kinship between the main caregiver and the child; age, gender, schooling and race/skin color of the main caregiver; number and description of the people with whom the child or adolescent lives; main income earner; family income; type of housing; characteristics of the house (own, rented, transferred, occupied, street situation); number of rooms; and type of material (coated masonry, trimmed wood, coated rammed earth, uncoated rammed earth, reused wood, straw, other).

### Data collection

Data collection was in charge of some of the research team members, Scientific Initiation and scholarship fellows and of the project. The collection procedure was standardized and all the subjects were previously trained with the objective of ensuring data reliability.

### Period

The data collection process was developed from May to July 2019.

### Sample

The sample of people invited to take part in the study was non-probabilistic and followed the convenience method. The number of black- and brown-skinned children and adolescents monitored by the CAPS-IJ when collecting the data for this study was 264 (total n). It was possible to establish contact with 96 family members, who were invited to take part in individual and face-to-face interviews.

Considering the different barriers faced, such as low income, difficulty in urban mobility, school holidays, lack of social support network to share care and changes in telephone numbers and addresses, among others, frequency during the data collection period was low.

A total of 55 interviews were carried out with family members of black-skinned children and adolescents monitored by the Brasilândia CAPS-IJ, excluding six because they were outside the scope and/or for lacking correct identification, resulting in 49 interviews. There were four interviews with mothers of two children; in other words, 49 interviews with 47 family caregivers.

The sample represents 18.5% of the population of 264 children and adolescents, which points to a limitation because it can render the results less conclusive, and shows that the profile of the people responsible for the care of the black-skinned population monitored by the CAPS-IJ corroborates with the inequalities of gender, race and class of the relationship between the caregiver and the social context in which the care takes place.

### Data treatment and analysis

The continuous variables were described using mean values or medians and standard deviations. The categorical variables were described resorting to absolute and relative frequencies. To assess whether the distribution of the variables differed according to self-declared race/skin color, conservative hypothesis tests were performed taking into account the sample size, which was evaluated as statistically reliable. The Student’s t test was used for the continuous variables. In the case of the categorical ones, Fisher’s Exact test was employed^(20)^. The analyses were performed with the aid of the R 3.5.1 software^(21)^. A 5% significance level was considered for the hypothesis tests.

In order to understand the sociofamily profile of black-skinned children and adolescents with mental health problems and their main caregivers, the concept of intersectionality was used as an analytical tool through which it was intended to understand the way in which oppression and vulnerabilities overlap, through complex intersections that structure gender, race and class positions in the social fabric^(22)^.

Intersectionality investigates the way in which intersectional power relations influence social relationships, as well as the individual experiences in everyday life. As an analytical tool, it considers that the “race”, “class”, “gender”, “sexual orientation”, “disabilities”, “nationality”, “ethnicity” and “age group” categories, among others, are inter-related and jointly structured. “Intersectionality is a way to understand and explain the complexity of the world, of people and of human experiences”^(23)^.

### Ethical aspects

The study followed the ethical and legal precepts set forth in Resolutions 466/2012^(18)^ and 510/2016^(19)^ of the National Health Council. It was approved by the Permanent Committee on Ethics and Research with Human Beings of the Nursing School at the University of São Paulo under CAAE 68987317.0.0000.5392 and opinion No. 2,444,763; as well as by its counterpart from the São Paulo Municipal Health Department under CAAE 68987317.0.3001.0086 and opinion No. 2,619,218. All the participants were clarified regarding conduction of the study and signed the Free and Informed Consent Form.

## Results

Table1 presents the results related to the gender and kinship degree of the caregivers with the children and adolescents, which shows predominance of women responsible for the care of black-skinned girls, most of them monitored by the CAPS-IJ.

**Table 1 -:** Profile corresponding to black-skinned children and adolescents monitored in the Brasilândia CAPS-IJ^*^-II. São Paulo, SP, Brazil, 2019

Variables	Female (n=44)	Male (n=5)	Total (n=49)	p
User’s gender				
Female	42/44 (95.5%)	1/5 (20%)	43/49 (87.8%)	<0.001
Male	2/44 (4.5%)	4/5 (80%)	6/49 (12.2%)	
Kinship with the user				
Mother	39/44 (88.6%)	0/5 (0%)	39/49 (79.6%)	<0.001
Father	0/44 (0%)	4/5 (80%)	4/49 (8.2%)	
Paternal grandmother	1/44 (2.3%)	0/5 (0%)	1/49 (2%)	
Brother/Sister	1/44 (2.3%)	1/5 (20%)	2/49 (4.1%)	
Others	3/44 (6.8%)	0/5 (0%)	3/49 (6.1%)	
The interviewee is the main caregiver (Yes)	39/44 (88.6%)	4/5 (80%)	43/49 (87.8%)	0.495
Kinship between caregiver and user				
Mother	39/44 (88.6%)	0/5 (0%)	39/49 (79.6%)	<0.001
Father	0/44 (0%)	5/5 (100%)	5/49 (10.2%)	
Paternal grandmother	2/44 (4.5%)	0/5 (0%)	2/49 (4.1%)	
Others (former sister-in-law, cousin, aunt, godmother)	3/44 (6.8%)	0/5 (0%)	3/49 (6.1%)	

^*^Psychosocial Care Center for Children and Adolescents

Table2 below presents the caregivers’ characteristics related to gender, schooling and skin color. Women caregivers, who are 84.1% black-skinned, present a mean age of 39.7 years old. The male interviewees are black-skinned, their mean age is 42.6 years old and are all illiterate in terms of schooling.

**Table 2 -:** Caregivers’ schooling levels according to gender, Brasilândia CAPS-IJ^*^. São Paulo, SP, Brazil, 2019

Variables	Female (n=44)	Male (n=5)	Total (n=49)	p
Caregiver’s age; Mean ± SD	39.7 ± 9.9	42.6 ± 6.4	40 ± 9.6	0.527
Caregiver’s schooling level				
Illiterate	2/44 (4.5%)	0/5 (0%)	2/49 (4.1%)	0.740
Incomplete Elementary School	13/44 (29.5%)	2/5 (40%)	15/49 (30.6%)	
Complete Elementary School	5/44 (11.4%)	0/5 (0%)	5/49 (10.2%)	
Incomplete High School or Technical Education	1/44 (2.3%)	0/5 (0%)	1/49 (2%)	
Complete High School or Technical Education	16/44 (36.4%)	2/5 (40%)	18/49 (36.7%)	
Incomplete Higher Education	5/44 (11.4%)	0/5 (0%)	5/49 (10.2%)	
Complete Higher Education	2/44 (4.5%)	1/5 (20%)	3/49 (6.1%)	
Caregiver’s skin color				
Black	14/44 (31.8%)	2/5 (40%)	16/49 (32.7%)	1.000
Brown	23/44 (52.3%)	3/5 (60%)	26/49 (53.1%)	
White	6/44 (13.6%)	0/5 (0%)	6/49 (12.2%)	
Indigenous	1/44 (2.3%)	0/5 (0%)	1/49 (2%)	

^*^Psychosocial Care Center for Children and Adolescents

Table3 presents the household characteristics according to the caregivers’ gender and shows that children and adolescents lived, on average, with another 3.9 individuals in houses with approximately 3.6 rooms. Also differentiating by the caregivers’ gender: 3.5 rooms among female caregivers and 4.2 in households where the caregivers are men.

When the main caregiver is female, 38.6% of the children and adolescents also live with their father, whereas when the main caregiver is male, 80% also live with their mothers. It is noted that women provide care by themselves, whereas men, when in charge, also count on women’s participation.

**Table 3 -:** Characteristics of the houses according to the caregivers’ gender, Brasilândia CAPS-IJ^*^. São Paulo, SP, Brazil, 2019

Variables	Female (n=44)	Male (n=5)	Total (n=49)	p
Number of people living in the same house as the child; Mean ± SD	3.9 ± 1.3	3.6 ± 0.9	3.9 ± 1.3	0.620
The child lives with his/her mother (Yes)	36/44 (81.8%)	4/5 (80%)	40/49 (81.6%)	1.000
The child lives with his/her father (Yes)	17/44 (38.6%)	5/5 (100%)	22/49 (44.9%)	0.014
The child lives with a brother of his/hers (Yes)	26/44 (59.1%)	4/5 (80%)	30/49 (61.2%)	0.636
The child lives with grandparents (Yes)	6/44 (13.6%)	0/5 (0%)	6/49 (12.2%)	1.000
Family income; Median [Quartiles]	1,100 [998 - 1,800]	1,900 [1,400 - 2,500]	1,150 [998 - 1,975]	0.142
Income source - Wages	26/44 (59.1%)	5/5 (100%)	31/49 (63.3%)	0.143
Income source - Help from relative	2/44 (4.5%)	0/5 (0%)	2/49 (4.1%)	1.000
Income source - Benefits^†^	25/44 (56.8%)	3/5 (60%)	28/49 (57.1%)	1.000
Type of house				
House	38/43 (88.4%)	3/5 (60%)	41/48 (85.4%)	0.055
Village house	5/43 (11.6%)	1/5 (20%)	6/48 (12.5%)	
Apartment	0/43 (0%)	1/5 (20%)	1/48 (2.1%)	
Characteristics of the house				
Own	15/44 (34.1%)	2/5 (40%)	17/49 (34.7%)	0.897
Rented	15/44 (34.1%)	1/5 (20%)	16/49 (32.7%)	
Transferred property	10/44 (22.7%)	2/5 (40%)	12/49 (24.5%)	
Occupied	4/44 (9.1%)	0/5 (0%)	4/49 (8.2%)	
Number of rooms in the house; mean ± SD	3.5 ± 1.1	4.2 ± 1.3	3.6 ± 1.1	0.181
Type of material of the house				
Masonry with siding	43/44 (97.7%)	5/5 (100%)	48/49 (98%)	1.000
A masonry room and another one made of wood	1/44 (2.3%)	0/5 (0%)	1/49 (2%)	

^*^Psychosocial Care Center for Children and Adolescents; ^†^Social benefit, retirement, LOAS, pension, unemployment insurance

An important fact is that 9.1% of the female caregivers live in conditions of housing insecurity, in precarious “occupations”.

The family income mostly comes from wages, for all the male caregivers and for 59.1% of their female counterparts. This fact indicates an important gender-based inequality in accessing the formal labor market. We can infer that women adapt their participation in the labor market based on the care need, in addition to the sexist barriers inherent to the capitalist system. Another important income source among female caregivers are the benefits (LOAS), *Bolsa Família*, retirement, pension and unemployment insurance.

Table4 details the characteristics according to race/skin color as analysis category, among female caregivers, 85.7% of whom are black-skinned.

**Table 4 -:** Characteristics according to the caregivers’ race/skin color, Brasilândia CAPS-IJ^*^. São Paulo, SP, Brazil, 2019

Variables	Black (n=16)	Brown (n=26)	White (n=6)	Indigenous (n=1)	p
Caregiver’s age; mean ± SD	39.6 ± 9.9	39.4 ± 9.1	41.8 ± 11.5	51 (n=1)	0.656
Caregiver’s gender (Male)	2/16 (12.5%)	3/26 (11.5%)	0/6 (0%)	0/1 (0%)	1.000
Caregiver’s schooling level					
Illiterate	1/16 (6.2%)	0/26 (0%)	0/6 (0%)	1/1 (100%)	0.394
Incomplete Elementary School	3/16 (18.8%)	9/26 (34.6%)	3/6 (50%)	0/1 (0%)	
Complete Elementary School	1/16 (6.2%)	4/26 (15.4%)	0/6 (0%)	0/1 (0%)	
Incomplete High School or Technical Education	1/16 (6.2%)	0/26 (0%)	0/6 (0%)	0/1 (0%)	
Complete High School or Technical Education	7/16 (43.8%)	9/26 (34.6%)	2/6 (33.3%)	0/1 (0%)	
Incomplete Higher Education	1/16 (6.2%)	3/26 (11.5%)	1/6 (16.7%)	0/1 (0%)	
Complete Higher Education	2/16 (12.5%)	1/26 (3.8%)	0/6 (0%)	0/1 (0%)	

^*^Psychosocial Care Center for Children and Adolescents

A caregiver declared herself indigenous, female and illiterate, data that intersectionally show the vulnerability in access to education among women from indigenous peoples.

In the variable that indicates who the children and adolescents live with, the presence of grandparents in the households is significantly higher in the homes where the caregivers are white-skinned, followed by those where the caregiver is brown-skinned, and only 6.2% of the children live with grandparents in houses where the caregiver is self-declared black-skinned. In the home of the only indigenous caregiver interviewed, the child lives with her mother and brother, and the family lives exclusively on social benefits (*Bolsa Família*).

Table5 presents the characteristics of the houses and the income sources according to the participants’ race/skin color. The data also revealed that some users live simultaneously with people of different degrees of kinship, regardless of self-declared race/skin color. In relation to income, the table presents the calculation of the median and its origin according to race/skin color.

**Table 5 -:** Characteristics of the houses according to the caregivers’ race/skin color, Brasilândia CAPS-IJ^*^. São Paulo, SP, Brazil, 2019

Variables	Black (n=16)	Brown (n=26)	White (n=6)	Indigenous (n=1)	p
Number of people living in the same house as the child; mean ± SD	3.9 ± 1.5	3.8 ± 1.3	4.2 ± 1.2	Three	0.844
The child lives with his/her mother (Yes)	11/16 (68.8%)	23/26 (88.5%)	5/6 (83.3%)	1/1 (100%)	0.420
The child lives with his/her father (Yes)	6/16 (37.5%)	13/26 (50%)	3/6 (50%)	0/1 (0%)	0.807
The child lives with a brother of his/hers (Yes)	10/16 (62.5%)	13/26 (50%)	6/6 (100%)	1/1 (100%)	0.101
The child lives with grandparents (Yes)	1/16 (6.2%)	3/26 (11.5%)	2/6 (33.3%)	0/1 (0%)	0.378
Family income; Median [Quartiles]	1,139 [993.5-2,625]	1,150 [998-1,975]	1,000 [627-1,100]	1,200 [1,200-1,200]	0.750
Income source - Wages	10/16 (62.5%)	17/26 (65.4%)	4/6 (66.7%)	0/1 (0%)	0.771
Income source - Help from relative	0/16 (0%)	1/26 (3.8%)	1/6 (16.7%)	0/1 (0%)	0.370
Income source - Benefits^†^	10/16 (62.5%)	14/26 (53.8%)	3/6 (50%)	1/1 (100%)	0.912
Type of housing					
House	15/16 (93.8%)	19/25 (76%)	6/6 (100%)	1/1 (100%)	0.596
Village house	1/16 (6.2%)	5/25 (20%)	0/6 (0%)	0/1 (0%)	
Apartment	0/16 (0%)	1/25 (4%)	0/6 (0%)	0/1 (0%)	
Characteristics of the house					
Own	4/16 (25%)	12/26 (46.2%)	1/6 (16.7%)	0/1 (0%)	0.699
Rented	6/16 (37.5%)	7/26 (26.9%)	2/6 (33.3%)	1/1 (100%)	
Transferred property	5/16 (31.2%)	5/26 (19.2%)	2/6 (33.3%)	0/1 (0%)	
Occupied	1/16 (6.2%)	2/26 (7.7%)	1/6 (16.7%)	0/1 (0%)	
Number of rooms in the house; mean ± SD	3.8 ± 1	3.6 ± 1.3	2.8 ± 0.8	3	0.371
Type of material of the house					
Masonry with siding	16/16 (100%)	25/26 (96.2%)	6/6 (100%)	1/1 (100%)	1.000
A masonry room and a wooden room	0/16 (0%)	1/26 (3.8%)	0/6 (0%)	0/1 (0%)	

^*^Psychosocial Care Center for Children and Adolescents; ^†^Social benefit, retirement, LOAS, pension, unemployment insurance

Of the total, 99.05% live in brick-lined houses, and a small percentage of brown-skinned caregivers have a wooden room. Among the black-skinned female caregivers, 25% own their house and the percentage is 46.2% among the brown-skinned ones, showing stratification by skin color in access to housing rights. Looking at the territory, around 10% of all caregivers live in occupation conditions, 20% live in transferred housing, 35% in their own homes and 35% in rented places.

In the “income source” variable corresponding to the caregivers interviewed, the mean is approximately R$ 1,100.00, close to one minimum wage, an amount that does not differ significantly in the race/skin color variable of the sample. According to the income source, the Social Security benefits are as relevant as wages among black-skinned caregivers, whereas wages are less relevant for brown- and white-skinned caregivers. We understand that benefits are received when they are not associated with wages, as they are for children and, thus, especially women, the majority among caregivers and curators, cannot have a formal job to supplement their income. As for help from relatives, which is related to the social support network, among white-skinned people it is present in 16.7%, with lower percentages among brown- and black-skinned individuals (3.8% and 0%, respectively), a situation that aggravates the contractual condition of black-skinned families.

## Discussion

The division and appreciation of care work in society’s production mode and capitalist organization is perpetuated unevenly. Gender, race and class inequalities, intersected, impose on black-skinned women the work of sustaining life, social reproduction, caring for children, adolescents, the elderly and household chores, with a significant impact on well-being, due to overloads and invisibility of their own needs^(24)^.

The results support the patriarchal and sexist organization of families which, despite the feminist movement, does not present significant changes in the division of household tasks between men and women^(25)^. They are responsible for the care work, which is invisible and unpaid, almost entirely depending on other women^(26 27)^.

When the woman is the main caregiver, her quality of life is affected by lower schooling and remuneration levels, by informal and precarious work relationships and by limited access to specialized services, which results in higher morbidity and mortality rates, as well as with higher exclusion rates the darker the skin color^(28)^.

Considerably differentiated by gender, family income renders the discussion about gender inequality in accessing the labor market indispensable, as it is women that suffer the inequalities in terms of income and contractual power. Above all, black-skinned women, historically victims of slave labor, remain at a disadvantage in terms of access to formal work. The Brazilian “aesthetic model” of white privileges is highlighted, in the tradition of the “matriarchy of misery”, which allocated few labor rights to black-skinned women, who are the majority of domestic workers^(29)^.

Black- and brown-skinned women are the majority in informality, without social security rights or a “formal contract”, with lower wages and in menial jobs, with accumulation of working hours, in addition to adjusting their participation in the labor market, based on the needs regarding time for child and family care^(30 32)^.

Deconstruction of the patriarchal, racist and capitalist society has been one of the goals of the black feminist movement since the 1970s; however, racial differences between women have hardly diminished. The concept of intersectionality has its history rooted in the life experiences of black-skinned women, and not only concerns the different dimensions of identity but the interactions between them^(33)^.

In the struggle for the right to work, it was black-skinned women that did not have access to day care for their own children^(34)^. It is in the intersectionality between race, gender and class, aggregated as social markers that the inequalities between black- and white-skinned women are better understood^(23 35)^.

The debate of “blackening the feminist agenda” and replacing issues with the black movement began at the end of the 20^th^ century and extends into contemporary times. With the rise of black feminism, the racism that affects women, who have always been at the forefront of struggles for racial and gender equality, becomes explicit^(36 37)^.

According to the National Household Sample Survey (*Pesquisa Nacional por Amostra de Domicílios*, PNAD), there are 11.6 million families consisting only of women and their children^(38)^. There is a phenomenon called “loneliness of black-skinned women”, who mostly assume their children and remain alone, with the inequalities amplified when the children have mental health problems^(26)^.

The constitution of white and brown families with more social support network than in families of black-skinned people configures a system of discrimination by skin color, also called colorism. In colorism, women with darker skin are more discriminated against, have a smaller network, social place and access to rights, and black-skinned women are more vulnerable^(39)^.

Unequal access to education is a consequence of a system marked by interconnected oppressions, an aspect that needs to be analyzed intersectionally according to gender, race/skin color, social class and ethnicity. This reality was questioned at the 2001 III Conference against Racism and Xenophobia, to guarantee access to work and higher education for black-skinned women^(40)^.

In relation to the condition of indigenous women, access to education is restricted, they end up “condemned to a cycle of poverty, fewer opportunities and lack of conditions for decision-making” and, according to the results, they are also made vulnerable with a small social support network^(41)^.

It is evidenced that gender, race, social class and ethnic inequalities, as well as situations of domestic violence, work devaluation, food insecurity, income and housing instability and absence of a social support network are social determinants of mental health^(42)^. Some authors point to “racism and denial of rights” as “a high-potential intrinsic factor in psychological distress”^(43)^. The international literature on intersectional inequalities in mental health shows that research is still limited in volume and not methodologically structured. However, it highlights the value of an intersectional analysis of the inequalities between population groups for establishing priorities in practices and policies^(44)^.

With the “gender” variable as a social marker for understanding the health of women responsible for taking care of people with mental health problems, overload, low quality of life and dissatisfaction with family relationships are identified^(45)^. By intersecting race/skin color and class, social inequalities are verified, which, when overlapped, maintain cycles of violence, oppression and power and structure the complex social fabric^(16)^.

In the current situation, we face intensification of the dismantling of the SUS, of the National Mental Health Policy, and also of the PNSIPN, with ordinances and projects that trigger serious setbacks, ignoring democratically conquered rights. Necropolitics has been institutionalized in Brazil since the legal-parliamentary coup of 2016 and has worsened since 2018; it includes psychiatric hospitals with overfunded beds, compulsory hospitalizations in therapeutic communities and violence as a norm, as well as scrapping of the Psychosocial Care Network^(46)^. This reality is aggravated by the neglect in management of the COVID-19 pandemic in Brazil, which is not based on evidence and worsens the social, racial and gender inequalities^(16)^.

When discussing the sociofamily profile of black-skinned children and adolescents monitored by a CAPS-IJ, from the perspective of intersectionality, there is an evident need to take into account the diversity of the population, the accumulation of oppressions and vulnerabilities, and the individuality of each child and/or adolescent and their caregivers. We hope that this research may contribute to the teams of community and territorial services from the Health Care Networks, in the elaboration of strategies with families, to care for, share, support and alleviate the barriers encountered by black-skinned women, from the perspective of access to and guarantee of human rights.

The study has limitations in the size and scope of the sample: people linked to only one Psychosocial Care Center, in a given territory of the city of São Paulo, Brazil. It is necessary to expand evidence in studies that contribute to ensuring health care equality and comprehensiveness in the SUS.

For the theoretical-practical advancement in the Health area, especially in Nursing, we hope that the reflections may contribute to the intersectional understanding of equality in care practices, as well as in the critical analysis of work organization as a professional category, where direct care is usually associated with low recognition, low schooling levels, gender, race/skin color and social class.

The sociopolitical factors evidence the need for tools to include race, gender and class in the care management and in public policies as categories of analysis, in order to combat multiple levels of violence and violation of rights, such as structural racism and sexism, throughout the health care system. We consider the need to disseminate scientific evidence in the field of Mental Health Nursing in the intersection between gender, race and social class.

## Conclusion

The research results revealed that those responsible for the care of black-skinned children and adolescents monitored by the CAPS-IJ are almost entirely women, black-skinned (black or brown) mothers or grandmothers, with unequal access to education, work and housing, which are constitutional social rights in Brazil. This study elucidates that, in families where the caregivers are black-skinned women, they are more vulnerable because have less contractuality and fragile social networks, and accumulate the challenges of caring for black-skinned children and adolescents with mental health problems.

The concept of intersectionality used as a theoretical and methodological tool proposes thinking about the structural inseparability of racism, capitalism and cisheteropatriarchy, and the resulting articulations, which repeatedly make black-skinned women more exposed and vulnerable. Among other different social markers gender, race, class and sexuality interact with each other, structuring life in society.

Investigating the living conditions of black-skinned children and adolescents with mental health problems has the social function of identifying and qualifying the fight against inequalities. The research presents a reflection grounded on the gender, race/skin color and class inequalities and points out that it is necessary to consider the subjects in their social, family and cultural contexts. Through intersectionalities, it can be understood how racism and sexism impact on people’s health, and strategies that guarantee equality and integrality in mental health care can be devised.
